# The Effect of Axial Traction MRI on the Articular Cartilage Visibility in Thumb Carpometacarpal Arthritis

**DOI:** 10.7759/cureus.52025

**Published:** 2024-01-10

**Authors:** Akira Ikumi, Yuichi Yoshii, Sho Kohyama, Sho Iwabuchi, Takeo Mammoto, Takeshi Ogawa, Masashi Yamazaki

**Affiliations:** 1 Department of Orthopedic Surgery, Institute of Medicine, University of Tsukuba, Ibaraki, JPN; 2 Department of Orthopedic Surgery and Sports Medicine, Tsukuba University Hospital Mito Kyodo General Hospital, Ibaraki, JPN; 3 Department of Orthopedic Surgery, Tokyo Medical University Ibaraki Medical Center, Ibaraki, JPN; 4 Department of Orthopedic Surgery, Kikkoman General Hospital, Chiba, JPN; 5 Department of Orthopedic Surgery, National Hospital Organization, Mito Medical Center, Ibaraki, JPN

**Keywords:** cartilage defect, articular cartilage, magnetic resonance imaging, axial traction, thumb carpometacarpal arthritis

## Abstract

Objectives: Thumb carpometacarpal arthritis has a high incidence. However, the degree of damage to the cartilage has not been accurately assessed. The purpose of this study was to examine the effects of axial traction of the thumb carpometacarpal joint during magnetic resonance imaging (MRI) on the visibility of articular cartilage in patients with thumb carpometacarpal arthritis and to evaluate the articular cartilage defect using MRI findings.

Materials and methods: Forty-four patients with thumb carpometacarpal arthritis (14 males, 30 females) and a mean age of 67.3±8.6 years were classified according to Eaton Stages 1, 2, 3, and 4 in 2, 14, 24, and 4 patients, respectively. Axial traction MRI was performed with and without traction (3 kg) using 3-Tesla MRI (Siemens Magnetom Skyra) with a 3D T2* multiecho data imaging combination. The effectiveness of traction was verified using the joint space width before and after traction at five points (central, volar, dorsal, radial, and ulnar margins) and the original articular cartilage outline visibility classification (poor, intermediate, complete). The rate of remaining cartilage on each joint surface was also evaluated. Statistical significance was set at p<0.05 in this study.

Results: Joint space width increased significantly at all points with traction (P<0.01). The grade of articular cartilage outline visibility significantly improved from seven intermediate and 37 poor cases to 15 complete, 23 intermediate, and six poor cases (P<0.01). Significantly more articular cartilage remained in Stages 1-2 compared with Stages 3-4 arthritis of both articular surfaces (P<0.01 in first metacarpal, P=0.01 in trapezium).

Conclusion: Axial traction of the thumb increased the joint space width and improved articular cartilage visibility in the thumb carpometacarpal joint. Our results suggested that axial traction MRI can be used for noninvasive evaluation of articular cartilage defects in patients with thumb carpometacarpal arthritis and aid in selecting the optimal surgical procedure.

## Introduction

The thumb carpometacarpal joint is a saddle joint that connects the first metacarpal (MC1) and the trapezium and can be moved in multiple directions during daily activities such as pinching or grasping because of its anatomical characteristics [1,2]. The incidence of thumb carpometacarpal arthritis is high, occurring in >15% of adults aged >30 years and one-third of postmenopausal women, despite it being a non-weight-bearing joint [3-6]. Thumb carpometacarpal arthritis is usually diagnosed based on patient history, physical examination, and radiographs. The Eaton classification of thumb carpometacarpal arthritis is widely used to determine the staging and severity of this type of arthritis [7,8]. However, the degree of damage to the articular cartilage has not been accurately assessed, because the Eaton classification is based only on radiographs. One study reported that the intra- and inter-examiner reliabilities of this classification are low [9], whereas other studies have reported a poor correlation between clinical symptoms and intraoperative articular cartilage findings [10,11].

Magnetic resonance imaging (MRI) is widely used to evaluate articular cartilage damage. However, accurate articular cartilage evaluation of the thumb carpometacarpal joint is challenging because of its anatomical complexity and relatively small size compared to those of large joints such as the hip and knee. Although some reports have evaluated the articular cartilage of the thumb carpometacarpal joint using MRI [12-14], an accurate method of evaluation has not yet been established because of the contact of the articular cartilage between the MC1 and the trapezium, as well as the underestimation of the degree of cartilage damage compared with other pathological findings [15].

To enhance the visibility of the articular cartilage, we performed an MRI while applying axial traction to the thumb carpometacarpal joint in healthy volunteers [16]. The joint space width of the thumb carpometacarpal joint was significantly increased in accordance with the traction weight, and the articular cartilage visibility of the thumb carpometacarpal joint was significantly improved by axial traction. This method of applying axial traction to improve the visualization of articular cartilage has also been previously used to observe other joints, such as the elbow and knee [17,18].

The aim of this study was to examine the effects of axial traction during MRI of the thumb carpometacarpal joint on the visibility of articular cartilage and to evaluate the remaining articular cartilage in patients with thumb carpometacarpal arthritis.

## Materials and methods

Study population

This study was approved by the institutional review board of the Tsukuba University Hospital Mito Clinical Education and Training Center, Ibaraki, Japan (No. R04-01), and was performed in accordance with the ethical standards laid down in the 1964 Declaration of Helsinki and its later amendments.

We enrolled 44 patients who visited our facility between April 2021 and October 2022 and were diagnosed with thumb carpometacarpal arthritis. Written informed consent was obtained from each patient after a thorough explanation of the objectives, methods, and expected complications.

Image acquisition

**Figure 1:**
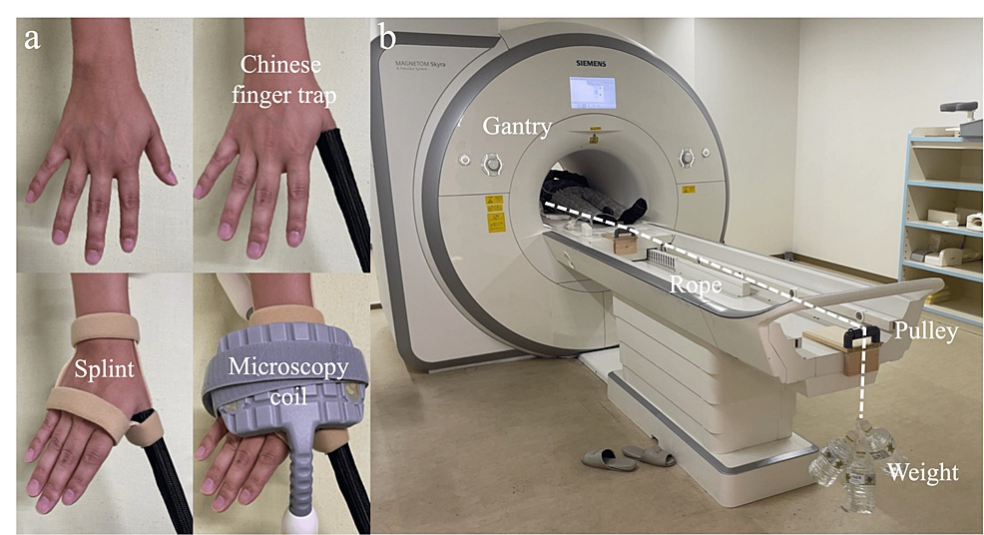
Application of axial traction during thumb carpometacarpal joint MRI (a) The thumb is enclosed within a Chinese finger trap. Then, the wrist and thumb are fixed using a custom-made splint. Finally, the microscopy coil is placed around the wrist. (b) The Chinese finger trap is connected to the traction weight using a nonelastic rope routed through the pulley system.

We used a 3-Tesla (3T) whole-body MRI system (Magnetom Skyra, Siemens Healthneers AG©, Munich, Germany) with a 4-channel 3T special purpose coil (Siemens Healthneers AG©, Munich, Germany). For the MRI sequence, a three-dimensional T2* multiecho data imaging combination (MEDIC) scan was used with the following parameters: slice thickness, 0.2 mm; slice gap, 0.15 mm; field of vision, 130 × 130 × 78 mm; matrix, 384 × 292; time to repeat, 20 ms; echo time, 11.0 ms; and flip angle, 25°. The required time, according to the protocol, was 5 min and 43 sec for each image. The patients were asked to lay supine on a table with their arms outstretched and their forearms pronated at the side of the body. The wrist and thumb were fixed with custom-made splints to standardize the limb position during the MRI examination. The hand under observation was centered parallel to the long axis of the gantry (Figure 1a).

Application of axial traction during MRI

The patient’s thumb was enclosed within a Chinese finger trap (Allen® Sterile Mesh Finger Traps, AliMed, Inc., Massachusetts, USA) using a rope. After fixing the wrist and thumb using a splint to keep the thumb position at 40-degree abduction during traction, the other end of the rope was hung over the edge of the MR table via a pulley system and attached to non-magnetic traction weights (Figure 1b). The MRI was initially performed without traction (no weight was used), followed by an MRI with traction. A traction weight of 3 kg was used based on our previous research [16].

Image analysis

We evaluated the effects of traction on the joint space width and articular cartilage outline visibility. In this study, the joint space width was defined as the space between opposing articular cartilages within the target joint. All MR images were independently evaluated by two orthopedic surgeons (with 15 and 10 years of clinical experience, respectively). All study images were interpreted on a workstation (Materialise Mimics, version 20.0; Materialise©, Leuven, Belgium), which was used to obtain the multiplanar reconstructed (MPR) images. Specifically, coronal and sagittal images were reconstructed parallel to the longitudinal axis of the first metacarpal region. The plane connecting the depressed portions of the distal metacarpal condyles with respect to the long axis was defined as coronal, and the plane perpendicular to the coronal plane was defined as sagittal. This procedure was performed by the first author for all the images. The images were initially enlarged, and the grayscale contrast was adjusted to optimize the visualization of the assessed structure. The images were then randomly numbered to minimize bias.

Measurement of the joint space width

**Figure 2:**
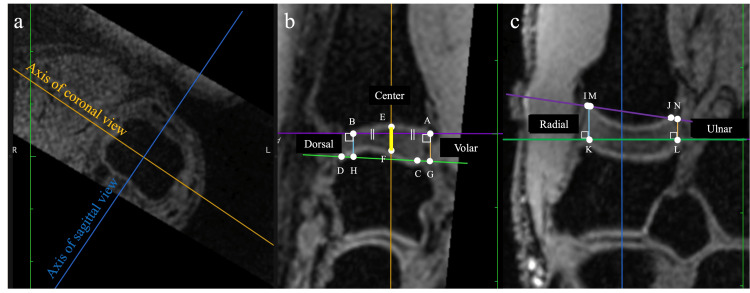
Definition of measurement points. (a) The coronal and sagittal axes are defined using the axial plane at the first metacarpal bone head. (b) Sagittal image of first carpometacarpal joint. Distances E-F, A-G, and B-H were defined as the center, volar, and dorsal distances, respectively. (c) Coronal image at the first carpometacarpal joint. Distances K-M and L-N were defined as the radial and ulnar distances, respectively.

Joint space width was measured on sagittal and coronal images at the center of the proximal articular surface of the first metacarpal bone as previously described [16]. On the sagittal image, the AB line, the line through both the volar (point A) and dorsal (point B) borders at the proximal articular surface of the first metacarpal bone; and the CD line, the line through both the volar (point C) and dorsal (point D) borders of the distal articular surface of the trapezium were drawn first. Subsequently, a perpendicular line was drawn at the center of the AB line and the intersection point of the articular surface of the first metacarpal bone was labeled as point E and the intersection point of the articular surface of the trapezium was labeled as point F. Furthermore, the intersection points of the perpendicular line drawn from points A and B to the CD line were labeled as points G and H. On the sagittal image, the following two lines were drawn: the IJ line, the line through the radial (point I) and ulnar (point J) borders of the proximal articular surface of the first metacarpal bone, and the KL line, the line through the radial (point K) and ulnar (point L) borders at the distal articular surface of the trapezium. Subsequently, the intersection points of the perpendicular lines drawn from points K and L to the IJ line were defined as points M and N. The distance between points E and F was defined as the center of the joint space width, those between points A and G and B and H were defined as the volar and dorsal joint space widths, and those between points I and M and J and N were defined as the radial and ulnar joint space widths, respectively (Figure 2).

Assessment of the articular cartilage outline visibility

**Figure 3:**
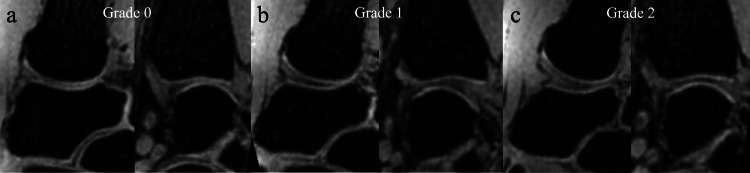
Articular cartilage outline visibility grade. (a) Grade 0 (poor), (b) Grade 1 (intermediate), (c) Grade 2 (complete).

The articular cartilage outline visibility was graded using a three-grade scale as previously described [16]. Grade 2 (complete) occurred when 100% of the articular cartilage outline was clearly visible in the entire range when facing the opposing articular cartilage. Grade 1 (intermediate) occurred when ≥50% but <100% of the articular cartilage outline was clearly visible in the range when facing the opposing articular cartilage. Grade 0 (poor) occurred when the articular cartilage outline was visible in <50% of the entire range when facing the opposing articular cartilage (Figure 3).

Measurement of the remaining cartilage rate

**Figure 4:**
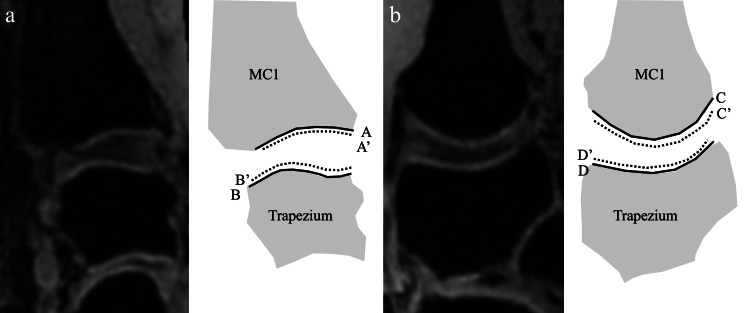
Calculation method of the remaining cartilage rate. (a) Sagittal image, (b) coronal image. The remaining cartilage (%) was calculated by dividing the distance of remaining cartilage (A’ to D’) by subchondral bone (A to D). A, B, C, D: the distance of subchondral bone, A', B', C', D': the distance of remaining cartilage, MC1: first metacarpal.

The rate of the remaining cartilage was evaluated using the same images on which joint space width was measured. On the sagittal and coronal images, the distance between the subchondral bone of the articular surface (line A-D) and the remaining cartilage (line A’-D’) was measured both on the first metacarpal bone and trapezium. The rate of the remaining cartilage (%) was calculated by dividing the distance of the remaining cartilage by subchondral bone, and the average value of the sagittal and coronal images was used as the remaining cartilage rate of each bone (Figure 4).

Statistical analyses

GraphPad Prism 8 (GraphPad Software, La Jolla, CA, USA) was used for all statistical analyses. All data were tested for normal distribution using the Shapiro-Wilk test. None of the data on articular cartilage outline visibility, joint space width, and remaining cartilage rate followed a normal distribution, owing to the small sample size. Therefore, the Mann-Whitney and Friedman tests were used to assess the differences in joint space widths and articular cartilage outline visibility with and without traction and the differences in the remaining cartilage rate in Stages 1-2 and Stages 3-4 patients. Spearman’s rank correlation coefficient was used to evaluate the correlation between the Eaton classification and articular cartilage visibility and changes in joint space width. Statistical significance was set at p <0.05. 

## Results

The study population comprised 44 patients (men: 14, women: 30), with a mean age of 67.3 ± 8.6 (range, 50-82) years. According to the Eaton classification, 2 patients were in Stage 1, 14 in Stage 2, 24 in Stage 3, and 4 in Stage 4. There is no patient who suspended the MRI examination due to discomfort relating to traction.

**Figure 5:**
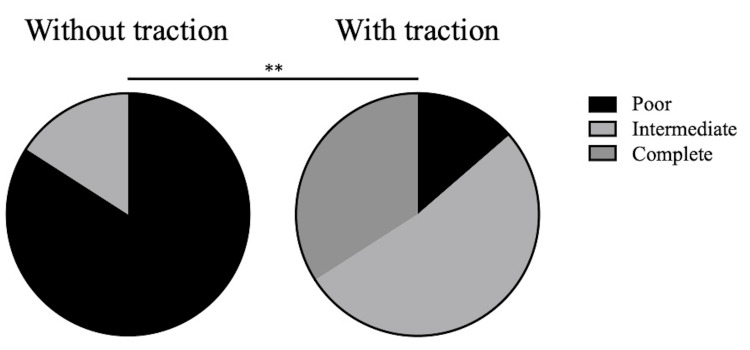
Visibility of the articular cartilage outlines. The visibility is significantly improved by traction. ** P < 0.01.

The grades of articular cartilage outline visibility were intermediate in 7 and poor in 37 cases without traction, complete in 15, intermediate in 23, and poor in 6 cases with traction. The visibility of the articular cartilage outlines significantly improved with traction (P<0.01) (Figure 5). There was a significant correlation between the Eaton classification stage and the grade of articular cartilage visibility with traction (r = -0.3096, P = 0.0409), but no correlation was observed without traction (r = -0.2890, P = 0.0571). 

**Figure 6:**
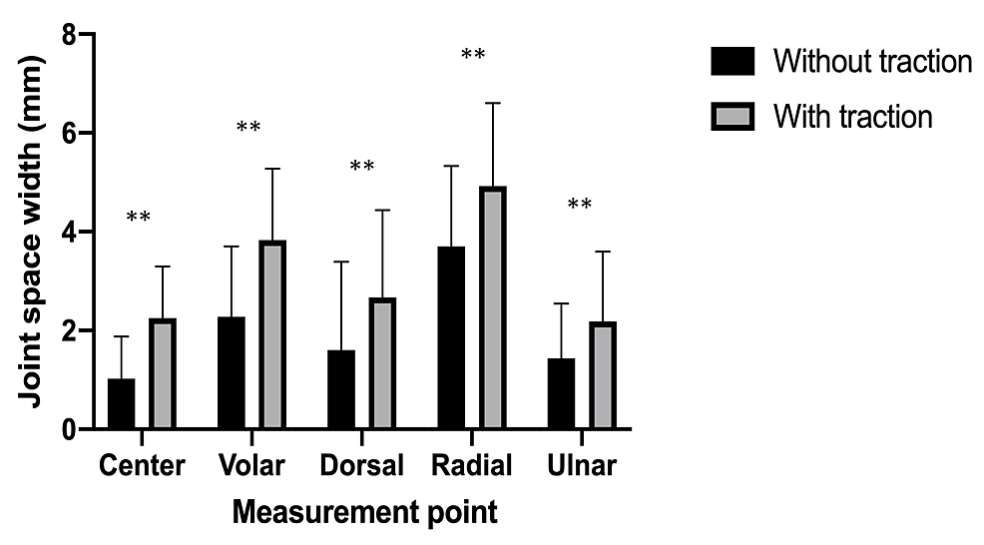
Joint space widths at each point (mean ± standard deviation). Joint space widths significantly widened after axial traction at all points. ** P < 0.01.

The joint space width without/with traction was 1.02 ± 0.85/2.25 ± 1.04 mm in the center, 2.28 ± 1.41/3.83 ± 1.43 mm in the volar edge, 1.60 ± 1.77/2.67 ± 1.75 mm in the dorsal edge, 3.70 ± 1.61/4.93 ± 1.66 mm in the radial edge, and 1.43 ± 1.10/2.18 ± 1.39 mm in the ulnar edge. The joint space width increased significantly at all points with traction (P < 0.01) (Figure 6).

The amount of change in joint space width before and after traction at each point was 1.23 ± 0.75 mm in the center, 1.55 ± 1.13 mm in the volar edge, 1.06 ± 1.11 mm in the dorsal edge, 1.22 ± 1.21 mm in the radial edge, and 0.75 ± 1.07 mm in the ulnar edge. No correlation between the Eaton classification stage and the change in joint space width was observed at any point (P=0.8801 at the center, 0.4884 at the volar, 0.6458 at the dorsal, 0.6080 at the radial, and 0.2983 at the ulnar edges). After comparing each point, the change in the ulnar edge was significantly smaller than that in the center (P<0.01), volar edge (P<0.01), and radial edge (P=0.02). 

**Figure 7:**
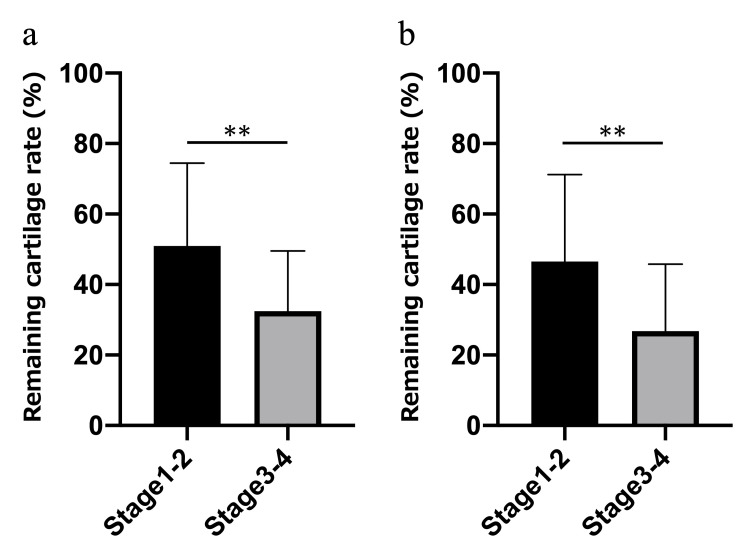
Remaining cartilage rate on sagittal and coronal images. (a) Sagittal image; (b) coronal image. The presence of cartilage remained significant in Eaton Stages 1-2. ** P<0.01.

The rate of remaining cartilage on the first metacarpal surface was 51.0 ± 22.8% in Stages 1-2 and 32.5 ± 16.8% in Stages 3-4 (P<0.01). The rate of remaining cartilage on the trapezium surface was 46.5 ± 23.9% in Stages 1-2 and 26.8 ± 18.7% in Stages 3-4 (P=0.01) (Figure 7). However, when considering individual cases, large cartilage defect (>50%) was observed in four Stages 1-2 patients (25.0% of all Stages 1-2 patients), and >50% cartilage remained intact in three Stages 3-4 patients (10.7% of all Stage 3-4 patients).

## Discussion

Our results showed that the visibility of the cartilage outline of the thumb carpometacarpal joint on MRI was significantly better when axial traction was applied to the thumb in patients with thumb carpometacarpal arthritis, similar to that in healthy volunteers. The rate of remaining cartilage was significantly higher in patients in the early Eaton stages. 

Various surgical procedures have been reported on thumb carpometacarpal arthritis [19]. For patients with mild osteoarthritic changes (Eaton classification Stages 1-2), ligament repair, arthroscopic synovectomy, and first metacarpal osteotomy are generally selected for joint-sparing surgery; whereas for patients with advanced osteoarthritic changes (Eaton classification Stages 3-4), ligament reconstruction with or without tendon interposition, arthrodesis, and artificial joint replacement are generally selected as non-joint-sparing surgery. Although the clinical outcomes of each surgical procedure are generally good [20], some joint preservation surgeries have achieved good symptomatic improvement even in patients with advanced osteoarthritic changes on plain radiographs [21,22]. In the present study, although the articular cartilage defect was significantly larger in patients with Eaton Stages 3-4, the cartilage remained >50% intact in 10% of those patients. The change in load distribution due to the first metacarpal osteotomy is one of the mechanisms used to reduce pain in patients with thumb carpometacarpal arthritis [23,24]. We believe that if the residual articular cartilage can be evaluated preoperatively, joint-sparing surgery can be performed more aggressively even in progressive cases. Thus, articular cartilage evaluation using axial traction MRI has the potential to be useful for surgeons in selecting less-invasive surgical procedures. We also believe that joint-sparing surgery is preferable for younger patients or those involved in heavy labor, although the risk of osteoarthritic progression exists even after long-term joint-sparing surgery. 

In this study, the widening of the articular surface due to axial traction was significantly smaller on the ulnar side. The anterior oblique (or volar beak) and dorsoradial ligaments have been reported as key ligaments in thumb carpometacarpal arthritis [25-27]. These ligaments contribute to the stability of the radiodorsal side of the thumb carpometacarpal joint. According to the progression of thumb carpometacarpal arthritis, loosening of these ligaments related to radiodorsal instability of the thumb carpometacarpal joint may have caused the difference in joint space width between the measurement points by traction in this study. Although our results suggest that the degree of traction-induced widening of the joint space can be used to evaluate joint instability, which reflects ligament dysfunction, the sample size was insufficient for evaluating joint instability (ligament dysfunction). Future research efforts should aim to increase the sample size and verify whether axial traction MRI can evaluate ligament function and articular cartilage. 

Badia et al. reported a treatment algorithm based on the intraoperative thumb carpometacarpal arthroscopy findings in 2006 [11]. Depending on the degree of articular cartilage damage or loss, joint-sparing or non-joint-sparing surgery was selected for this algorithm. This algorithm is more innovative than the Eaton classification because it assesses articular cartilage damage and loss with higher accuracy before deciding on the surgical technique. However, several limitations have been encountered, including the inability to evaluate the articular cartilage preoperatively, invasiveness, and the need to decide the surgical technique intraoperatively. Our results demonstrate that axial traction MRI of the thumb carpometacarpal joint could be used to preoperatively evaluate the articular cartilage condition, which would allow the selection of the optimal surgical technique that reflects the articular cartilage condition, rather than depending on the Eaton classification.

**Figure 8:**
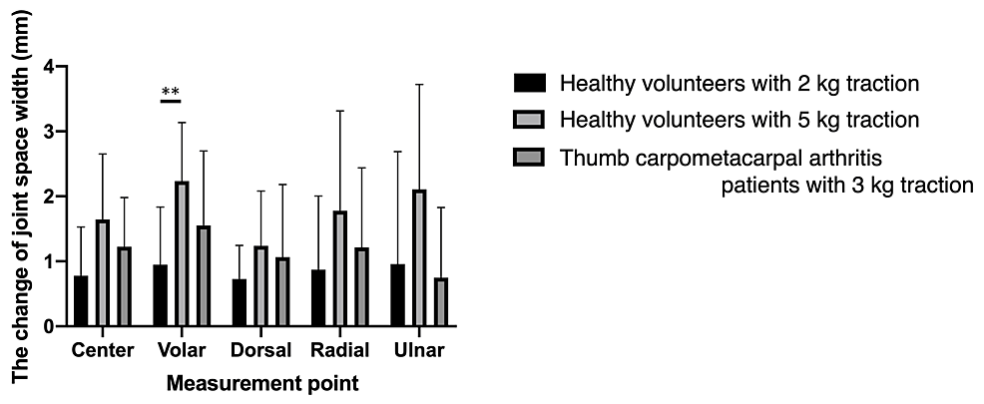
The change of joint space width between with and without traction. There is no significant difference between 3 kg traction in thumb carpometacarpal arthritis patients and both 2 kg and 5 kg traction in healthy volunteers at any measurement point by two-way ANOVA with the Geissler-Green house correction. ** P < 0.01 between 2 kg and 5 kg traction in healthy volunteers by Tukey’s multiple comparisons test.

This study has several limitations. First, the assessment of the rate of remaining cartilage was limited to a single slice of sagittal and coronal images in this study. Developing an evaluation method encompassing the whole joint surface, possibly using techniques such as 3D reconstruction, is necessary for a more accurate assessment of the articular cartilage condition in future studies. Second, the optimal traction weight was not evaluated in this study. While increasing the axial traction on the thumb with heavier weights might further widen the joint space and improve the articular cartilage outline visibility, this added traction may induce pain in these individuals. Compared with our previous result of the change in joint space width in healthy volunteers [16], there was no significant difference between 3 kg traction in patients with thumb carpometacarpal arthritis and 2 kg and 5 kg traction in healthy volunteers (Figure 8). Thus, a 3 kg traction was considered sufficient to evaluate articular cartilage defects in the thumb carpometacarpal joint. Thirdly, this study did not verify whether articular cartilage was correctly detected by MRI. To evaluate the precision of detecting articular cartilage through axial traction MRI, a study comparing the consistency between MRI and arthroscopy findings is necessary for future research. Finally, the axial traction system used in this study could not control the rotational force on the carpometacarpal joint of the thumb. A slight twist caused by axial traction may have affected the measurement of the joint space widths at each point. Therefore, the addition of a system to control the rotational force during axial traction becomes imperative.

## Conclusions

Axial traction of the thumb increased the joint space width and improved the visibility of the articular cartilage of the thumb carpometacarpal joint on the MRIs of patients with thumb carpometacarpal arthritis.

Our results suggest that axial traction MRI can be used to noninvasively evaluate articular cartilage defects in patients with thumb carpometacarpal arthritis.

Axial traction MRI holds potential as an evaluation index for selecting the optimal surgical method in patients with thumb carpometacarpal arthritis.
